# Expanded Newborn Screening for Inborn Errors of Metabolism in Hong Kong: Results and Outcome of a 7 Year Journey

**DOI:** 10.3390/ijns10010023

**Published:** 2024-03-11

**Authors:** Kiran Moti Belaramani, Toby Chun Hei Chan, Edgar Wai Lok Hau, Matthew Chun Wing Yeung, Anne Mei Kwun Kwok, Ivan Fai Man Lo, Terry Hiu Fung Law, Helen Wu, Sheila Suet Na Wong, Shirley Wai Lam, Gladys Ha Yin Ha, Toby Pui Yee Lau, Tsz Ki Wong, Venus Wai Ching Or, Rosanna Ming Sum Wong, Wong Lap Ming, Jasmine Chi Kwan Chow, Eric Kin Cheong Yau, Antony Fu, Josephine Shuk Ching Chong, Ho Chung Yau, Grace Wing Kit Poon, Kwok Leung Ng, Kwong Tat Chan, Yuen Yu Lam, Joannie Hui, Chloe Miu Mak, Cheuk Wing Fung

**Affiliations:** 1 Metabolic Medicine Unit, Department of Paediatrics and Adolescent Medicine, Hong Kong Children’s Hospital, Hong Kong, China; kiranm@ha.org.hk (K.M.B.);; 2 Newborn Screening Laboratory, Department of Pathology, Hong Kong Children’s Hospital, Hong Kong, China; 3 Clinical Genetics Service Unit, Hong Kong Children’s Hospital, Hong Kong, China; 4 Department of Paediatrics and Adolescent Medicine, Hong Kong Children’s Hospital, Hong Kong, China; 5 Department of Paediatrics and Adolescent Medicine, Tuen Mun Hospital, Hong Kong, China; 6 Department of Paediatrics and Adolescent Medicine, Queen Elizabeth Hospital, Hong Kong, China; 7 Department of Paediatrics and Adolescent Medicine, Princess Margaret Hospital, Hong Kong, China; 8 Department of Paediatrics, The Chinese University of Hong Kong, Hong Kong, China; 9 Department of Paediatrics, Prince of Wales Hospital, Hong Kong, China; 10 Department of Paediatrics and Adolescent Medicine, Queen Mary Hospital, Hong Kong, China; 11 Department of Paediatrics and Adolescent Medicine, United Christian Hospital, Hong Kong, China; 12 Department of Paediatrics and Adolescent Medicine, Pamela Youde Nethersole Eastern Hospital, Hong Kong, China; 13 Department of Paediatrics and Adolescent Medicine, Kwong Wah Hospital, Hong Kong, China

**Keywords:** newborn screening, inborn errors of metabolism, inherited metabolic disorders, dried blood spots, tandem mass spectrometry, Hong Kong

## Abstract

Newborn screening (NBS) is an important public health program that aims to identify pre-symptomatic healthy babies that will develop significant disease if left undiagnosed and untreated. The number of conditions being screened globally is expanding rapidly in parallel with advances in technology, diagnosis, and treatment availability for these conditions. In Hong Kong, NBS for inborn errors of metabolism (NBSIEM) began as a pilot program in October 2015 and was implemented to all birthing hospitals within the public healthcare system in phases, with completion in October 2020. The number of conditions screened for increased from 21 to 24 in April 2016 and then to 26 in October 2019. The overall recruitment rate of the NBS program was 99.5%. In the period between October 2015 and December 2022, 125,688 newborns were screened and 295 were referred back for abnormal results. The recall rate was reduced from 0.26% to 0.12% after the implementation of second-tier testing. An inherited metabolic disorder (IMD) was eventually confirmed in 47 infants, making the prevalence of IMD in Hong Kong 1 in 2674. At the time of the NBS result, 78.7% of the newborns with IMD were asymptomatic. There were two deaths reported: one newborn with methylmalonic acidemia cobalamin B type (MMACblB) died after the initial crisis and another case of carnitine palmitoyltransferase II deficiency (CPTII) died at 18 months of age after metabolic decompensation. The most common IMD noted were disorders of fatty acid oxidation metabolism (40%, 19 cases), closely followed by disorders of amino acid metabolism (38%, 18 cases), with carnitine uptake defect (19.1%, 9 cases) and citrullinemia type II (17%, 8 cases) being the two most common IMD picked up by the NBSIEM in Hong Kong. Out of the all the IMDs identified, 19.1% belonged to diverse ethnic groups. False negative cases were reported for citrullinemia type II and congenital adrenal hyperplasia during this period.

## 1. Background

Newborn screening (NBS) was pioneered by Robert Guthrie in the early 1960s [1]. In the 60 years since then, NBS has expanded to include dozens of inherited metabolic disorders (IMD), genetic and other disorders from a single blood spot using tandem mass spectrometry (TMS). In Hong Kong, NBS began with cord blood testing for congenital hypothyroidism and glucose-6-phosphate dehydrogenase (G6PD) deficiency in March 1984. NBS for congenital hearing loss was added in 2007 [2]. The expanded NBS using TMS was gaining momentum worldwide in the early 2000s as a health-effective and cost-effective intervention [3]. Thus, in 2013, Hong Kong set up a workgroup to review the evidence for the expansion of NBS for inborn errors of metabolism (NBSIEM) in Hong Kong. This workgroup had representatives from the Food and Health Bureau of HKSAR, the Department of Health, and the Hospital Authority and included disciplines of clinical genetics, pediatrics, obstetrics, pathology, public health, administrative medicine and information technology [3]. In 2015, a task force was established to plan and prepare for the implementation of a pilot study for NBSIEM. Selection of the IMD conditions to be included in the NBSIEM program were based on four agreed upon criteria: (1) screening capability—availability of accurate, reliable screening, diagnostic testing, and laboratory capability; (2) clinical significance—seriousness and number of cases encountered in our locality; (3) availability of treatment—efficacy and/or effectiveness of the treatment; and (4) favorable outcome after early treatment—adequacy of the understanding of the natural history of the condition and its long-term outcome with early treatment. NBSIEM in Hong Kong using TMS within the public healthcare system ran as a pilot study from October 2015 to March 2017 [3]. This ran in parallel with the use of cord blood for screening congenital hypothyroidism and G6PD deficiency, as it does at the present time. The results of this successful pilot, which recruited 15,138 newborns, were published and showed that the incidence of IMD was 1 in 1682 in Hong Kong, which was much higher than the previously estimated local incidence of 1 in 4122 to 1 in 7580 [3,4]. Thus, from March 2017 onwards, the NBSIEM program was extended to all birthing hospitals within the public healthcare system in phases, with completion in October 2020. The number of conditions screened for increased from 21 to 24 in April 2016 and then to 26 in October 2019 (Table 1). Congenital adrenal hyperplasia (CAH) is one condition on our NBSIEM program that is an endocrine metabolic condition. It was included in our NBSIEM since it could be screened simultaneously on the blood spot card together with other classical IMD conditions. Second-tier testing, biochemical and/or genetic, was added for 15 of the 26 target conditions in phases to reduce false positives and false negatives. In this paper, we present our findings and experience of screening 125,688 newborns between October 2015 and December 2022.

## 2. Materials

### 2.1. Subjects, Eligibility, and Coverage

A total of 126,287 newborns born in the eight birthing hospitals within the public healthcare system of Hong Kong between October 2015 and December 2022 were eligible to be recruited to the NBSIEM program. In the pilot phase I between October 2015 and March 2016, eligibility was defined as babies ≥34 weeks of gestation and birth weight >2000 g (g) from two birthing hospitals within the public healthcare system. In phase II of the pilot, from April 2016 to the end of the pilot study in March 2017, all newborns (preterm and term) were eligible from the same two birthing hospitals. Since the regularization of the NBSIEM, between March 2017 and October 2020, the eligibility was extended to include all newborns (preterm and term) from all eight birthing hospitals within the public healthcare system in phases. From October 2021 onwards, babies born before arrival were also eligible for NBSIEM. Video and pamphlet education were provided during both the antenatal and postnatal period to help parents understand the NBSIEM. Newborns were recruited only after written consent was obtained from the parents. Newborns born in the private sector were outside the scope of the current program running within the public healthcare system. Here, we focus on the results obtained from the 125,688 newborns who were recruited to the NBSIEM program between October 2015 and December 2022. Although data from the pilot program have been published before [3], we have included newborns from the pilot program and focused on aspects that the previous report did not focus on.

### 2.2. Data Collection

Data regarding the demographic and clinical conditions of the newborns were collected for this retrospective review, including sex, gestational age, presence or not of consanguinity, presence or not of symptoms at the time of the NBS result, biochemical and/or genetic diagnoses, and if death occurred due to their pathology. 

### 2.3. Sample Collection

The recommended time for blood sampling by heel prick after birth was between 24 and 72 h after birth. In low-birth-weight (<2000 g), premature infants (<34 weeks) and sick neonates (those admitted to the neonatal intensive care unit), blood sample collection was repeated at day 28 of life or before discharge, whichever came earlier. For those who had a blood transfusion before the NBS blood collection, blood sampling was performed again 120 days after the last transfusion, mainly for galactose-1-phosphate uridyl transferase (GALT) enzyme measurement. 

The dried blood spots (DBS) from newborns recruited for NBSIEM were sent to a central NBS laboratory for processing, analytics, and reporting. The NBS laboratory operates 5 working days (from Monday to Friday) per week and also 1 and 2 days within 3 and 4–5 consecutive holidays respectively. The result turnaround time was 5 working days. The parents were only informed if action was required, such as the need for an additional sample or an abnormal result. The DBS cards are stored for at least 6 months.

### 2.4. Panel of Conditions Screened

Table 1 shows the panel of diseases screened. They are divided into four main categories: disorders of organic acid metabolism (OA), disorders of amino acid metabolism (AAD), disorders of fatty acid oxidation (FAOD), and other IMD.

## 3. Methods

### 3.1. First-Tier Biochemical Tests

Screening of OA, AAD, and FAOD commenced on 1 October 2015. Amino acids, acylcarnitines, and succinylacetone were detected by non-derivatized TMS method on the DBS sample with the following commercial kits: Neobase (Revvity, Turku, Finland) from 1 October 2015 to 31 August 2021, MassChrom (Chromsystems, Gräfelfing, Germany) from 1 September 2021 to 27 April 2022, and Neobase 2 (Revvity, Turku, Finland) from 28 April 2022 to 31 December 2022. 

Screening of the “other IMD” (Table 1) started on 1 April 2016. The biotinidase (BTD) activity, GALT activity, and 17-hydroxyprogesterone (17OHP) were measured by the DELFIA^®^ enzymatic and immune-assay of the Genetic Screening Processor (GSP) platform (Revvity, Turku, Finland). 

### 3.2. Second-Tier Biochemical Tests

Second-tier testing for CAH began on 1 April 2016, with detection of 17OHP, androstenedione (D4A), and cortisol on the original DBS sample by an in-house liquid chromatography–tandem mass spectrometry (LC-MS/MS) method [5,6]. Second-tier testing for methylmalonyl-coA acidemia (MMA), cobalamin C (CblC) deficiency, propionic acidemia (PA), and homocystinuria (HCU) was launched in January 2021, with the detection of the total homocysteine (tHCY), methylmalonic acid (MMA), and methylcitric acid (MCA) on the original DBS sample determined by the in-house LC-MS/MS method [7]. (Table 1).

### 3.3. Second-Tier Genetic Screening Tests

The second-tier genetic screening test was launched in September 2021 for simultaneous screening of six IMDs: citrullinemia type II (CITII), carnitine uptake deficiency (CUD), glutaric acidemia type I, beta-ketothiolase deficiency, multiple carboxylase deficiency, and 3-hydroxy-3-methylglutaryl-co A lyase deficiency. The tests were performed on the original DBS samples with a fully validated custom-designed AmpliSeq panel (Illumina, San Diego, CA, USA) performed on the iSeq 100 (Illumina, USA) platform with procedures as previously described [8]. The scope was extended to include two more conditions, biotinidase deficiency (BTD) and argininemia, using the same assay in October 2022. (Table 1)

### 3.4. Reporting

The first-tier biochemical target markers are tabulated in Table 1. A second-tier biochemical or genetic test was only added on the original DBS sample with an abnormal first-tier marker. All the first- and second-tier results were analyzed and reported by chemical pathologists with molecular genetic pathology expertise. Conditions with second-tier testing were classified into two recall protocols, high- and low-risk, based on the type and level of the increased/decreased diagnostic biomarkers and on the risk of metabolic decompensation for the suspected diagnosis. In the high-risk protocol, the result was flagged before second-tier testing, and the neonate was immediately referred for clinical evaluation, diagnostic confirmatory testing, and, when needed, specific treatments. In low-risk cases, the second-tier test was performed, and the neonate was only recalled if the second-tier result was abnormal. The turn-around time was maintained at 5 working days. All abnormal IMD results were recalled to metabolic pediatricians for follow-up. All abnormal CAH results were recalled to pediatric endocrinologists for follow-up.

### 3.5. Data Analysis

Descriptive statistics were applied. A confusion matrix was utilized for the performance evaluations. The Hardy–Weinberg equilibrium was used to determine carrier frequency when needed. 

## 4. Results

### 4.1. Baseline Characteristics and Analysis

In the 7-year, 2-month period between October 2015 and December 2022, 125,688 (99.5%) of the 126,287 eligible newborns born in the eight birthing hospitals within the public healthcare system in Hong Kong were recruited to NBS for IMD. A total of 134,471 specimens were processed throughout this specified period. This included first and second, as well as third specimens from babies who required multiple sampling. A total of 295 newborns (0.23%) were recalled due to an abnormal result, of which 47 were eventually diagnosed to have an IMD. Thus, the collective incidence of IMD for the conditions screened in Hong Kong is 1 in 2674. Secondary IMD conditions beyond the scope of the current NBS program and non-IMD cases, such as vitamin B12 deficiency, heterozygote carriers, transitional abnormalities, and maternal conditions, were categorized as false positive cases. The positive predictive value (PPV) for the diagnosis of an IMD was 15.9%. 

Of the 47 cases, 25 neonates were female (53.2%) and 22 neonates were male (46.8%). Nine (19.1%) of the cases were of non-Chinese ethnicity, with 5 (10.6%) of them having consanguineous parents. None of the IMD cases of Chinese ethnicity had consanguineous parents. 

**Figure 1:**
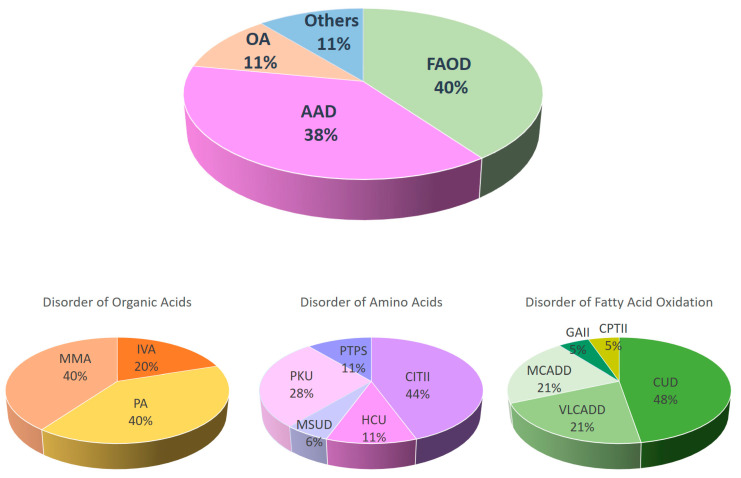
The percentage of different classes of IMD in the newborns screened by NBS from October 2015 to December 2022. OA = disorders of organic acid metabolism, AAD = disorders of amino acid metabolism, FAO = disorders of fatty acid oxidation, CUD = carnitine uptake defect, PKU = phenylketonuria, MCADD = medium-chain acyl-CoA dehydrogenase deficiency, VLCADD = very long-chain acyl-CoA dehydrogenase deficiency, CAH = congenital adrenal hyperplasia, MMA = methylmalonic acidemia, PA = propionic acidemia, HCU = homocystinuria, BTD = biotinidase deficiency, PTPS = 6-pyruvoyl-tetrahydropterin synthase deficiency, IVA = isovaleric acidemia, GAII = glutaric acidemia type II, CPTII = carnitine palmitoyltransferase II deficiency, MSUD = maple syrup urine disease.

**Figure 2:**
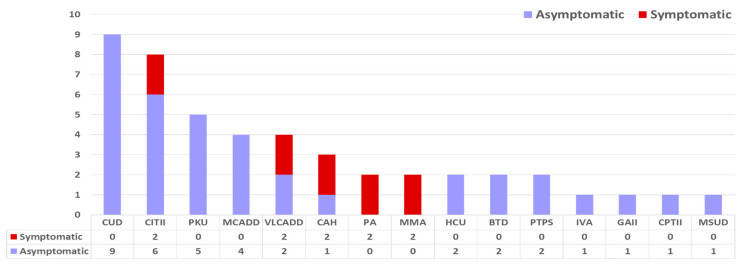
True positive cases N = 47 and their clinical condition at time of NBS. CUD = carnitine uptake defect, PKU = phenylketonuria, MCADD = medium-chain acyl-CoA dehydrogenase deficiency, VLCADD = very long-chain acyl-CoA dehydrogenase deficiency, CAH = congenital adrenal hyperplasia, MMA = methylmalonic acidemia, PA = propionic acidemia, HCU = homocystinuria, BTD = biotinidase deficiency, PTPS = 6-pyruvoyl-tetrahydropterin synthase deficiency, IVA = isovaleric acidemia, GAII = glutaric acidemia type II, CPTII = carnitine palmitoyltransferase II deficiency, MSUD = maple syrup urine disease.

The categories of IMD that are prevalent in Hong Kong are FAOD (40%, 19 cases; 1 in 6615) and AAD (38%, 18 cases; 1 in 6983), with similar incidences. OA (11%, 5 cases; 1 in 25,138) and other IMD (11%, 5 cases; 1 in 25,138) were less common (Figure 1 and Figure 2).

### 4.2. Disease Spectrum of IMD

For the 47 cases with IMD, a total of 15 different IMDs were detected, including five different FAOD and AAD each. Three different OA and two other IMDs were detected. Carnitine uptake defect (CUD) (19.1%, 9 cases) and citrullinemia type II (CITII) (17%, 8 cases) were the two most common IMD detected by NBS in Hong Kong, followed by phenylketonuria (PKU) (10.6%, 5 cases), medium-chain acyl-coA dehydrogenase deficiency (MCADD) (8.5%, 4 cases), and very long-chain acyl-coA dehydrogenase deficiency (VLCADD) (8.5%, 4 cases). The following conditions were detected in two newborns each (4.7%): congenital adrenal hyperplasia (CAH), methylmalonic acidemia (MMA), propionic acidemia (PA), homocystinuria (HCU), biotinidase deficiency (BTD), and 6-pyruvoyl-tetrahydropterin synthase deficiency (PTPS), whereas isovaleric acidemia (IVA), glutaric acidemia type II (GAII), carnitine palmitoyltransferase II deficiency (CPTII), and maple syrup urine disease (MSUD) were identified in one newborn each (2.1%) (Figure 1).

### 4.3. Outcome of the Newborns Screened by NBSIEM

At the time of the NBS result, 37 (78.7%) of the newborns with IMD were asymptomatic. However, 10 (21.3%) newborns with IMD already had developed symptoms before or at the time of the newborn screening result (2 CITII, 2 VLCADD, 2 CAH, 2 MMA, 2 PA) (Figure 2). All the OA cases identified by NBS (2MMA, 2 PA) were symptomatic before or at the time of the NBS result, with evidence of either poor feeding, metabolic acidosis, or hyperammonemia. One newborn with MMA cobalamin B type was born premature at 34 weeks’ gestation with a birth weight of 1.59 kg. He developed hyperammonemia, high anion gap acidosis, and lactic acidosis on day 2 of life. Although the NBS was performed on day 2 of life and was available on day 4 of life, he died on day 3 of life, before the NBS result was available. One MMAMut0 case and one PA case decompensated on day 3 of life with prompt treatment initiation, but the NBS result was available on day 5 of life for both these cases. Regarding the other PA case, the NBS result was available on day 4 of life, and the infant was noted to have hyperammonemia only when investigated for an abnormal NBS result. All the confirmed OA cases were detected before the implementation of second-tier biochemical testing. The MMAMut0 case has developed epilepsy as a complication of the condition but is thriving well otherwise at 7 years of age with normal development. Both the PA cases, aged 4 and 6 years at the time of this report, have feeding issues requiring gastrostomy feeding, developmental delay, and cardiomyopathy. Another newborn with VLCADD developed cardiac arrest at home at 56 h of life, requiring resuscitation before the NBS result was available on day 3 of life. However, after this initial crisis, with dietary therapy, she is thriving well at 32 months of age. The other VLCADD was well on day 3 of life at the time of the NBS result but had mildly elevated creatine kinase and mild cardiomyopathy when investigated. One CITII was born small for the gestational age and had significant clotting derangement on day 6 of life at the time of the NBS result. The other CITII case had cholestatic jaundice at the time of the NBS result on day 7 of life. Both these CITII cases were also detected before second-tier genetic testing was implemented. Both the CAH cases presented with impending salt-losing crisis with borderline low sodium and borderline high potassium at the time of the NBS result on day 5 and day 7, respectively. Both cases were identified after biochemical second-tier testing was implemented. One case with CPTII died at the age of 18 months after metabolic decompensation. 

The GAII baby picked up by NBS was initially flagged as a potential case of VLCADD because C14:1 was elevated alongside C6 to C14. He was initially started on a fat-restricted diet. However, he was switched back to a normal diet with high-dose riboflavin once rapid genetic sequencing identified two pathogenic variants in the *ETFDH* gene, which were B12-responsive. 

### 4.4. False Positives and Specificity

Five of the false positive cases were identified to have an IMD diagnosis beyond the scope of our current NBSIEM program. One newborn who had an elevated C3 on NBS in October 2018 was eventually diagnosed to have CblC deficiency, which was only added to our program in October 2019. Two newborns with elevated methionine were diagnosed to have the autosomal dominant form of methionine adenosyltransferase I/III deficiency. Both of them did not require any treatment. Another newborn with elevated C5 was diagnosed to have short-/branched-chain acyl-CoA dehydrogenase deficiency (SBCADD). This is considered a benign condition, and no treatment is needed. A newborn with persistent elevation of phenylalanine with negative genetic/biochemical testing for PKU and PTPS was subsequently diagnosed to have *DNAJC12* deficiency. 

Maternal conditions were also diagnosed in six of the false positive cases—one maternal PKU, two maternal CUD, and three 3-methylcrotonyl-CoA carboxylase deficiency. One father was diagnosed to have PKU during cascade family genetic screening of a baby diagnosed to have PKU by NBS. Both have mild PKU phenotypes only and do not require any treatment. 

We also had 53 call backs with abnormal C5DC (*n* = 28), C5OH (*n* = 10), GALT (*n* = 9), and arginine (*n* = 6), but none of these cases were found to have an IMD after further investigations. No abnormal call back for abnormal tyrosine or succinylacetone was reported during the period. 

The overall specificity of the NBS for IMD was 99.8%, with 248 tests being classified as false positive. The PPV of NBS for IMD was 15.9%. AAD and FAOD had the highest PPV of 20.2% and 17.8%, followed by OA (13.5%) and other IMD (12.5%). Table 2.

The majority of the current second-tier testing was incorporated into the NBS program before 31 December 2021. A subanalysis of the PPV and call backs before and after this date revealed that the PPV improved from 14.4% before 2022 to 33.3% in 2022. Similarly, the call back reduced by more than half, from 0.26% before 2022 to 0.12% in 2022.

### 4.5. False Negatives and Sensitivity

The NBSIEM program relies on a voluntary mechanism to report false negative results. However, 13 false negative cases of CITII and 3 cases of CAH were reported during this period. Four of the thirteen false negative cases of CITII were detected during a pilot second-tier screening for CITII. The remaining nine presented clinically with cholestasis, and one had undergone liver transplant before a diagnosis was made. Out of the nine missed cases, only two were missed after the implementation of second-tier genetic testing. 

Two of the three CAH cases presented salt-losing crisis at 1 and 7 months of age, respectively, whereas one case presented as the simple virilizing form at 18 months of age. A DBS sample was collected on D2 of life for all three cases of missed CAH. They were all full-term babies and had no exposure to steroids prior to collection. Two of the CAH cases who presented salt-losing crisis had an elevated 17-OHP of 25.4 nmol/L and 64 nmol/L, respectively (reference interval for full term < 25.0 nmol/L), by GSP. Second-tier testing for both showed a normal 17-OHP+ D4A/cortisol ratio of 0.37 and 1.4, respectively (reference < 2). For the simple virilizing case of missed CAH, the 17-OHP was normal by GSP, thus second-tier testing was not conducted. 

Inclusion of false negative cases in incidence calculation would make the incidence of CITII 1 in 5895 and that of CAH 1 in 20,948. Using the Hardy–Weinberg equilibrium, the carrier frequency of CITII in Hong Kong is estimated to be 1 in 37.

The overall sensitivity of NBS for IMD was 74.6%. The sensitivity for FAOD and OA disorders was 100%. The sensitivity of CITII and CAH was 58.1% and 62.5%, respectively, but for all other AAD and other IMD, it was 100% (Table 2).

## 5. Discussion

This study shows that 1 in 2674 newborns screened in Hong Kong has an IMD within the scope of the NBSIEM program. This incidence rate is similar to that described worldwide: China [9] (1:2548), Malaysia [10] (1:2916), Spain [11] (1:2670), Saudi Arabia [12] (1:2462), and Sweden [13] (1:3200). The incidence of IMD in Hong Kong is also comparable to that of congenital hypothyroidism, which is a cord-blood-based NBS program that has been running in Hong Kong since 1984 (1:2404) [14]. This indicates that although individually rare, the collective local incidence of IMD is much higher than the previously estimated incidence of 1 in 4122 to 1 in 7580 [3,4] based on retrospective data. 

However, the true collective incidence of IMD is Hong Kong is likely higher for several reasons. Firstly, if we include the five IMD conditions that we diagnosed but that are beyond the scope of our current NBSIEM program and the 16 false negative cases we missed, the incidence of IMD would be 1 in 1848. Secondly, during the period of October 2015 to December 2022, the total number of live births in Hong Kong, which includes babies born both within and outside the public healthcare system, was approximately 346,441 [15]. Therefore, these data are only representative of approximately 36.3% of all the live births in Hong Kong during the above said period. This number may also be affected by the fact that full implementation of NBSIEM in birthing hospitals within the public healthcare system was only achieved from October 2020 onwards. Thirdly, these data are only representative of 26 IMDs, which is only a tiny fraction of the ~1900 IMD conditions, thus largely underestimating the true incidence of IMD [16]. 

The overall PPV and recall rate of the NBS program stands at 15.9% and 0.23%. However, if we include the five IMD conditions beyond the scope of our NBS program and the six parental conditions we diagnosed, the PPV would be 19.7%. This PPV figure is similar to that of Singapore [17] (20%) and Germany [18] (11.3%), is much higher than that reported by eastern China [19] (1.7%) and Malaysia [10] (3.17%) and is lower than that reported by Spain [11] (24.6%), Norway [20] (41.2%), and Sweden [13] (47%). This discrepancy is likely due to the fact that the NBS program in Hong Kong and other Asian countries is relatively new compared to countries such as Norway and Sweden, who started using NBS TMS in 2012 and 2005, respectively. Another reason could be the different scope of conditions screened for in these countries. For example, China and Singapore, like Hong Kong, screen for CITII, whereas Spain, Norway, and Sweden do not screen for CITII [9,11,13,17,20]. Moreover, it was also noted that by the year 2022, our PPV had already increased to 33.3% and the recall rate had dropped to 0.12%. Apart from gaining experience, this improvement could be largely attributed to the addition of various second-tier testing methods that reduce the false positive rates and increase the specificity of the program. A similar improvement was noted in Singapore and Norway when they added second-tier testing [17,20]. 

The incidence of OA in this study was 1 in 25,138 which is similar to the incidence reported by Japan (1 in 22,000) and is a slightly higher incidence than that reported by Taiwan (1 in 18,000) [21]. Interestingly, China and Singapore have both reported a very high incidence of 1 in 8071 [9] and 1 in 6565 [17] for OA, respectively. This is because the latter two screening programs included an additional target condition in the OA category, namely, 3-methylcrotonyl-CoA carboxylase (3MCCC) deficiency, which can account for the seemingly higher incidence reported. Worldwide experts debate the benefit of screening for 3MCCC. In fact, New Zealand’s government did not observe any clinical benefit of screening for this condition and thus stopped screening for 3MCCC in 2015 [22]. 

All our PA and MMA patients were symptomatic at or before the time of the NBS result, despite NBS result being available before or on day 5 of life for all four cases. Grunert et al. [23] also found that 63% of their PA patients were symptomatic at the time of NBS reporting. Heringer et al. [24] previously reported that MMA and PA patients develop symptoms from the third to sixth day of life. Therefore, even if an NBS laboratory is operational seven days a week, some MMA and PA patients will still present clinically before the NBS results are available. Despite this, NBS is still believed to be a beneficial intervention to reduce time until diagnosis for organic acidemias and should be continued [24]. 

FAOD is the most common category of IMD in Hong Kong, with an incidence of 1 in 6615. However, this incidence was much higher than our other Asian counterparts’ reports, such as Japan (1 in 30,000) and Taiwan (1 in 34,000) [21]. Yunus et al. [10] in Malaysia also did not detect any FAOD in babies undergoing NBS and those who underwent high-risk screening of IMD, whereas China, as reported by both Men et al. and Deng et al. [9,19], had a midway incidence of FAOD between 1 in 10,917 to 1 in 11,145. Although Singapore reported an incidence of FAOD similar to Hong Kong (1 in 6330) [17], it may not be comparable to Hong Kong since the most common FAOD in Singapore is short-chain acyl coA dehydrogenase (SCAD) deficiency, which is not part of the Hong Kong NBS panel. Even though SCAD deficiency is a secondary target of the Recommended Uniform Screening Panel (RUSP), many states in the United States of America (USA), such as Michigan, stopped screening for this condition, which is believed to be a benign condition [25]. 

Keeping aside SCAD deficiency, most Asian countries, such as Singapore, Taiwan, and Hong Kong, report CUD to be the most common FAOD [17,21]. This is different from Western countries, such as Germany, Norway, and Italy, where MCAD is the most common FAOD [18,20,26]. Despite CUD being the most common FAOD in many Asian countries, the incidence of CUD was noted to appreciably higher in HK, being 1 in 13,965, when compared to China (1 in 20,285), Singapore (1 in 35,500), and Taiwan (1 in 70,000) [9,17,21]. Interestingly, although Deng et al. [9] reported the overall incidence of CUD in China to be 1 in 20,285, the incidence of CUD was highest (1 in 5549) in the southern part of China. Previously, Tang et al. [27] reported a founder mutation (NM_003060.4(*SLC22A5*):c.760C>T (p.Arg254Ter), which is prevalent in southern China. Hong Kong is also geographically situated in the southern part of China, which we assumed could account for this high incidence of CUD in our population. However, genetic analysis in our nine CUD cases showed that only case one was homozygous for this variant. The rest of our cases were compound heterozygous with other variants, including c.1400C>G and c.51C>G. Both of these pathogenic variants have been reported to be prevalent in the Chinese population in recent newborn screening studies emerging from China [28].

Regarding AAD, our incidence was 1 in 6983, with CITII (also known as citrin deficiency) being the most common AAD. Our incidence of AAD is much higher than that reported by Taiwan (1 in 17,000), Singapore (1 in 17,726), Japan (1 in 26,000), and Korea (1 in 29,000) [17,21]. Our incidence is comparable to that reported by China (1 in 5834) and Germany (1 in 5000) [9,21]. However, contrary to Hong Kong, PKU and not citrin deficiency is the most common AAD in both countries, with the incidence ranging from 1 in 5000 in Germany to 1 in 7854 in China [9,21]. Interestingly, PKU is also the commonest AAD reported by other Asian countries, such as Japan, Taiwan, and Singapore, with the incidence ranging from 1 in 46,000 to 1 in 138,000 [17,21], whereas Hong Kong has a PKU incidence of 1 in 25,137. The discrepancy in the incidence of PKU of other Asian countries when compared to China may be related to the genetic variation in the vast geographical region of China. Deng et al. reported that the incidence of PKU is highest in the northwest and southwest of China, with incidences ranging from 1 in 1356 to 1 in 5277 [9]. This part of China is connected by land to Europe and Russia and may have ancestral genetic links with Caucasians to account for the high incidence of PKU in this region, whereas southern China, as reported by Deng et al., has a PKU incidence of 1 in 20,380, resonating with our findings in Hong Kong [9]. 

In our review, CITII was the most frequent AAD detected by NBS in Hong Kong, with an incidence of 1 in 15,711, followed by PKU. This incidence is similar to the CITII detection rate by NBS reported by Taiwan (1 in 18,006) and that of the estimated disease frequency reported by Japan (1 in 17,000) [29,30,31]. However, our incidence of CITII is largely underestimated since the incidence was based on true positive cases only. Inclusion of false negative increases the incidence significantly to 1 in 5895, making CITII the most common IMD in Hong Kong. The estimated carrier frequency of CITII is 1 in 37. In 2019, Kabata et al. from Japan reported a comparable carrier frequency of CITII to be 1 in 33 [32]. In Hong Kong, the high incidence and carrier frequency may be attributed to the NM_014251.3(*SLC25A13*):c.852_855del (p.Met285fs) variant, which is prevalent in southern China as a result of the founder effect [33]. The genetic analysis of the 21 CITII cases we have identified, 8 of which were detected by NBS and 13 of which were missed cases, confirms this founder effect. Ten of our CITII cases were homozygous for the c.852_855del pathogenic variant, nine were heterozygous for this variant together with another heterozygous pathogenic/likely pathogenic variant, and only two did not harbor this variant.

It is postulated that newborns affected by the NICCD phenotype of CITII start to develop citrullinemia and cholestasis from day 5 of life onwards [34]. However, 25% of the CITII cases detected by us were symptomatic with either florid cholestasis or impending liver failure before or by day 5 of life. Recent studies have shown that CITII can affect the fetus because the mitochondria in the citrin-deficient fetus does not produce adequate energy, resulting in the baby having intrauterine growth retardation [34]. We postulate it is the same phenomenon that resulted in the early appearance of symptoms in two of our cases. Zhang et al. also reported acute liver failure resulting in death while awaiting liver transplant in an infant with citrin deficiency [35]. Therefore, call backs for suspected citrin deficiency should be treated with urgency and vigilance, especially if they are born small for the gestational age. 

In the majority of cases, newborns affected by CITII are asymptomatic at the time of NBS and some even have no clear amino acid and biochemical changes and do not develop NICCD at all, making it a disease that is easy to be missed by NBS. Previous local studies and NBS studies from Taiwan and Australia have all reported missing CITII [29,33,36]. To improve the sensitivity of NBS, both Hong Kong and Taiwan have added molecular second-tier testing for CITII with a cut-off of 25 µmol/L and 20 µmol/L, respectively, for second-tier testing [29]. After the addition of second-tier genetic testing in June 2018, Taiwan did not report any false negative cases of CITII until September 2021. However, in Hong Kong, we have identified two false negative cases with citrulline levels of 19 µmol/L and 20 µmol/L, respectively. While this can be due to the different first-tier cut-off adopted, this may also be attributed to the slow rise of citrulline in the first few days of life. 

Another condition that our NBS program missed was CAH. The aim of NBS is to detect the severe salt-wasting form of classical CAH. Therefore, missing the simple virilizing form of CAH is frequently reported in the literature. [37,38,39]. However, in addition to one simple virilizing CAH, we missed two salt-losing classical CAH despite second-tier testing. This has been previously reported in NBS data from Wisconsin and Minnesota in the USA, where they reported missing ten and five cases of salt-wasting CAH and simple virilizing type CAH, respectively. [37,40,41] However, in response to this false negative salt-losing CAH, we have lowered our second-tier biochemical ratio of 17OHP + D4A/cortisol from 2.0 to 1.1 since 1 December 2020 and have not missed any cases of salt-wasting CAH since then. Nonetheless, it is of the utmost importance that clinicians do not delay effective life-saving treatment in cases with clinical suspicion even if infants have passed the NBS. Furthermore, addition of 21-deoxycortisol into the second tier testing panel was in progress.

During the period of this study, no argininosuccinic aciduria, citrullinemia type I, or tyrosinemia type I were detected. Japan and Singapore did not report any tyrosinemia type I either in previous NBS studies [17,21]. Albeit uncommon, China did report tyrosinemia type I with an incidence of 1 in 373,667 [9]. Regarding citrullinemia type I, the incidence reported is 1 in 199,000 in Taiwan, 1 in 253,559 in China, and 1 in 306,000 in Japan [9,21]. Argininosuccinic aciduria has a very low incidence in Asia (Taiwan (1: 593,000), China (1: 887,458), and Japan (1: 1,121,000) [9,21]. The low incidence of these two conditions is the likely reason we have not detected any cases yet, and it is only a matter of time before we identify these IMD in Hong Kong. 

We have successfully overcome the initial challenge of implementing an NBS program. The program is well accepted by the general public, as shown by our recruitment rate of 99.5%. This recruitment rate is much higher those reported by Singapore [17] (71%) and Denmark [42] (91.4%) and is comparable to the recruitment rate in Taiwan [43] (98%). Moreover, a local study conducted by Belaramani et al. [44] reported that 97.9% of parents who had participated in NBS for uncommon disorders found it useful. This shows that the local parents are generally satisfied with the newly implemented NBS program. 

In conclusion, NBS for IMD is a vital public health program that has successfully been implemented in Hong Kong since 2015. It has had a profound impact on the well-being of our newborns by successfully identifying a majority of the pre-symptomatic newborns, allowing for timely interventions to reduce mortality. Moreover, IMD is not as rare in Hong Kong as previously assumed. With advances in medical technologies, NBS is also evolving, and new diseases and new approaches are constantly being developed. We will continue evaluate the NBS program time and again to consider new second-tier testing, both biochemical/genetics, and new candidate conditions to add to the panel and perhaps remove conditions that are not reaping the intended benefits. 

## Figures and Tables

**Table 1:** Disorders, first-tier biochemical markers, and second-tier testing.

Disorders of Organic Acid Metabolism (8 Conditions)	Primary Marker/Ratio	Second-Tier Testing
Glutaric acidemia type I	↑ C5DC	Genetic (*September 2021 onwards*)
Isovaleric acidemia	↑ C5, ↑ C5/C2, ↑ C5/C3	
Methylmalonic acidemia	↑ C3, ↑C3/C2	Biochemical: MMA, MCA, tHCY by LC-MS/MS (*January 2021 onwards*)
Methylmalonic acidemia and homocystinaemia (cobalamin C deficiency) (*October 2019 onwards*)		
Propionic acidemia		
Multiple carboxylase deficiency	↑ C5OH, ↑ C5OH/C8	Genetic (*September 2021 onwards*)
Beta-ketothiolase deficiency	↑ C5:1, ↑ C5OH, ↑C5OH/C8	Genetic (*September 2021 onwards*)
3-hydroxy-3-methylglutaryl-coA lyase deficiency	↑ C5OH, ↑ C6DC, ↑C5OH/C8	Genetic (*September 2021 onwards*)
**Disorders of Amino Acid Metabolism (9 conditions)**		
Argininemia (*October 2019 onwards*)	↑ Arg	Genetic (*October 2022 onwards*)
Argininosuccinic acidemia	↑Cit	
Citrullinemia type I		
Citrullinemia type II		Genetic (*September 2021 onwards*)
Phenylketonuria	↑ Phe, ↑ Phe/Tyr Ratio	
6-pyruvoyl-tetrahydropterin synthase deficiency		
Homocystinuria	↑ Met, ↑ Met/Phe	Biochemical: tHCY by LC-MS/MS (*January 2021 onwards*)
Maple syrup urine disease	↑ Xle, ↑Valine	
Tyrosinemia type I	↑ Tyr, ↑Succinylacetone	
**Disorders of Fatty Acid Oxidation Metabolism (6 conditions)**		
Carnitine-acylcarnitine translocase deficiency	↑C16, C18, ↑C18:1, ↑C18:2, ↓C0/(C16+C18) ↑ (C16+C18:1)/C2	
Carnitine palmitoyltransferase II deficiency		
Carnitine uptake deficiency	↓ C0	Genetic (*September 2021 onwards*)
Glutaric acidemia Type II	↑ C4-C18	
Medium chain acyl-coA dehydrogenase deficiency	↑ C6, ↑ C8, ↑C10, ↑ C10:1, ↑C8/C10	
Very long-chain acyl-coA dehydrogenase deficiency	↑ C14, ↑ C14:1, ↑ C14:2, ↑ C14:1/C16, ↑C14:1/C2, ↑C14:1/C12:1	
**Other IMD (3 conditions)**		
Biotinidase deficiency (*April 2016 onwards*)	↓ BTD activity	Genetic (*October 2022 onwards*)
Classic galactosemia (*April 2016 onwards*)	↓ GALT activity	
Congenital adrenal hyperplasia (*April 2016 onwards*)	↑ 17OHP by GSP	Biochemical: 17OHP, D4A and cortisol by LC-MS/MS. Positive if elevated (17OHP +D4A)/cortisol ratio with elevated 17OHP (*April 2016 onwards*)

**Table 2:** Results of NBSIEM in Hong Kong: frequency of screened disorders and diagnostic specificity, sensitivity, and positive predictive value of the method.

Newborn Screened 125,688	True Positive	Incidence	False Positive	False Negative	Positive Predictive Value
			**Specificity (%)**	**Sensitivity (%)**	
**Total**	**47**	**1 in 2674**	248 * (99.8)	**16 (74.6)**	**15.9%**
**Disorders of Organic Acids**	5	1 in 25,138	32 (99.97)	0 (100)	13.5%
Isovaleric acidemia	1	1 in 125,688	3 (99.99)	0 (100)	25%
Methylmalonic acidemia (methylmalonyl-coA mutase deficiency)	2	1 in 62,844	29 (99.98)	0 (100)	12.1% ^#^
Propionic acidemia	2	1 in 62,844		0 (100)	
**Disorders of Amino Acid**	18	1 in 6983	70 (99.94)	13 (58.1)	20.5%
Citrullinemia type II	8	1 in 15,711 #	49 (99.96)	13 (38.1)	14.3% ^#^
Maple syrup urine disease	1	1 in 125,688	1 (99.99)	0 (100)	50%
Classic phenylketonuria	5	1 in 25,137	15 (99.98)	0 (100)	22.7%
6-pyruvoyl-tetrahydropterin synthase deficiency	2	1 in 62,844		0 (100)	
Homocystinuria	2	1 in 62,844	5 (99.99)	0 (100)	28.6%
**Disorders of Fatty Acid Oxidation**	19	1 in 6615	58 (99.93)	0 (100)	24.7%
Carnitine palmitoyltransferase II deficiency	1	1 in 125,688	10 (99.99)	0 (100)	9.1%
Carnitine uptake deficiency	9	1 in 13,965	42 (99.96)	0 (100)	16.6%
Glutaric acidemia type II	1	1 in 125,688	1 (99.98)	0 (100)	3.6%
Medium chain acyl-coA dehydrogenase deficiency	4	1 in 31,422	3 (99.99)	0 (100)	57.1%
Very long-chain acyl-coA dehydrogenase deficiency	4	1 in 31,422	2 (99.99)	0 (100)	57.1%
**Other IMD**	5	1 in 25,138	35 (99.97)	3 (62.5)	12.5%
Biotidinase deficiency	2	1 in 62,844	20 (99.98)	0 (100)	9%
Congenital adrenal hyperplasia	3	1 in 41,896 #	15 (99.99)	3 (50)	16.7%

* This figure includes the 53 call backs without any true positive cases. # This incidence is calculated from true positive cases only; false negative cases are not included in this calculation.

## Data Availability

The data supporting this study’s findings are available on reasonable request from the corresponding author.
